# Genome-Wide Identification and Expression Analysis of the U-Box E3 Ubiquitin Ligase Gene Family Related to Monoterpene Indole Alkaloids Biosynthesis in *Uncaria rhynchophylla*

**DOI:** 10.3390/ijms27125198

**Published:** 2026-06-09

**Authors:** Yu Zhou, Detian Mu, Yingying Shao, Xiaohua Huang, Haifeng Yan, Iain W. Wilson, Rongchang Wei, Lina Zhu, Xinghui Liu, Deyou Qiu, Qi Tang

**Affiliations:** 1 Yuelushan Laboratory, College of Horticulture, Hunan Agricultural University, Changsha 410128, China; 18574824089@163.com (Y.Z.); mudetian12580@163.com (D.M.); syy2250519718@126.com (Y.S.); zhulina_2021@163.com (L.Z.); 18627362607@163.com (X.L.); 2 Biotechnology Research Center, Xiangxi Academy of Agricultural Sciences, Jishou 416099, China; 3 Guangxi Academy of Agricultural Sciences, Nanning 530007, China; wt131913@163.com (X.H.); gstsyhf@163.com (H.Y.); 4 Commonwealth Scientific and Industrial Research Organisation (CSIRO) Agriculture and Food, Canberra, ACT 2601, Australia; lain.wilson@csiro.au; 5 State Key Laboratory of Tree Genetics and Breeding, Research Institute of Forestry, Chinese Academy of Forestry, Beijing 100091, China; qiudy@caf.ac.cn; 6 State Key Laboratory for Quality Ensurance and Sustainable Use of Dao-di Herbs, Beijing 100700, China

**Keywords:** *Uncaria rhynchophylla*, ABA treatment, expression analysis, U-box E3 ubiquitin ligase, monoterpene indole alkaloids

## Abstract

Plant U-box E3 ubiquitin ligases (PUBs) are involved in various biological processes in response to abiotic stress. Recent studies have shown that E3 ubiquitin ligases can regulate the production of important compounds in medicinal plants by targeted degradation of transcription factors. Abscisic acid (ABA), a plant stress response hormone, can cause changes in the content of the medically important monoterpene indole alkaloids (MIAs) in *U. rhynchophylla.* In this study, we explored the relationship between *UrPUB* gene expression and MIA content. ABA was applied to tissue-cultured seedlings of *U. rhynchophylla*, resulting in consistent changes in the content of four MIAs. Seventy-three *UrPUB* genes were identified and bioinformatically characterized from the genome sequence of *U. rhynchophylla*. The expression levels of transcription factors involved in regulating the biosynthesis pathway of MIAs and *UrPUB* genes with higher RNA transcript levels in the roots were determined. Co-expression association analysis revealed that *UrPUB17*, *UrPUB40*, *UrPUB41*, *UrPUB44* and *UrPUB55* negatively correlate with *UrGATA8* and *UrWRKY37* under ABA stimulation. Based solely on these correlation data, we hypothesize that these UrPUBs might regulate MIA biosynthesis via ubiquitination of UrGATA8 and UrWRKY37, but direct evidence (protein interaction, ubiquitination, degradation, or genetic manipulation) is lacking. This study identified the *UrPUB* gene family in the *U. rhynchophylla* genome and proposes this ubiquitination model as a testable hypothesis, not a demonstrated mechanism. These findings provide new insights into the biological function of the *PUB* family in response to ABA.

## 1. Introduction

**Figure 1:**
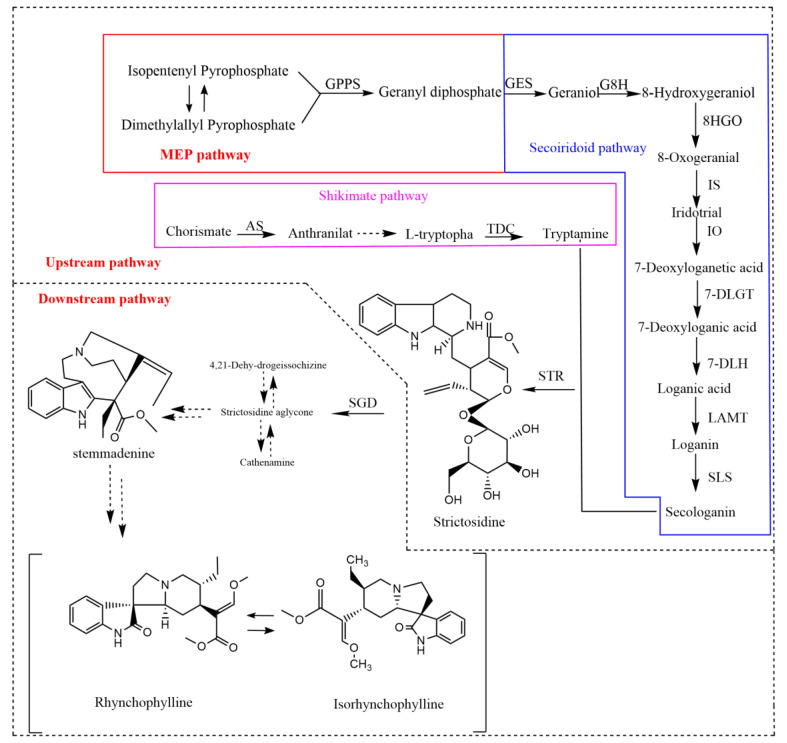
The known portion of the MIA biosynthesis pathway. The solid line represents a confirmed chemical reaction, and the dashed line represents a hypothetical reaction.

*Uncaria rhynchophylla* is a traditional Chinese medicine that has a long history of use for its heat-clearing, antihypertensive, and anticonvulsant properties [1]. Modern research indicates that the main active constituents in *U. rhynchophylla* have neuroprotective, hypotensive, and antidepressant effects. The major active medically important components are mainly monoterpenoid indole alkaloids (MIAs) [2,3,4]. The biosynthesis pathway of MIAs in *U. rhynchophylla* is similar to that in *Catharanthus roseus*, which serves as a model plant for studying MIAs as it has been more extensively investigated. The biosynthesis of MIAs follows a complex pathway, involving at least 30 coordinated enzymatic reaction steps, leading to the production of at least 35 known intermediates [5]. The known portion of the MIAs biosynthesis pathway is shown in Figure 1. The pathway can be divided into an upstream pathway and a downstream pathway. The upstream pathway includes the iridoid and tryptamine pathways. The secologanin produced by the upstream pathway and tryptamine are catalyzed by strictosidine synthase to produce strictosidine, which is then hydrolyzed by strictosidine β-D-glucosidase (SGD) to generate strictosidine aglycone for subsequent reactions [6]. In the pathway of *U. rhynchophylla*, *UrTDC* and *UrSTR* have been verified to possess corresponding catalytic functions through prokaryotic expression in vitro [7,8]. The biosynthesis pathways of secondary metabolites in medicinal plants are often regulated by certain transcription factors [9], and regulation of the biosynthesis of MIAs in *U. rhynchophylla* is also thought to be controlled by various transcription factors. The expression levels of *UrGATA7* and *UrGATA8* in *U. rhynchophylla* tissue culture seedlings under different light treatments are significantly correlated with the changes in pathway gene expression, suggesting their potential involvement in the regulation of alkaloid biosynthesis in *U. rhynchophylla* [10]. Yeast one-hybrid experiments and dual-luciferase assays have demonstrated that UrbHLH1 can bind to the promoters of *UrG10H* and *Ur10HGO* to regulate the biosynthesis of MIAs [11], and UrWRKY37 has also been shown to potentially regulate the biosynthesis of MIAs by binding to the promoter of *UrTDC* [12]. UrTCP4 might interact with the promoters of *UrLAMT* and *Ur7-DLH* [13]. When exploring the biosynthetic mechanisms of secondary metabolites in medicinal plants, it is necessary to consider both transcription factors and enzyme genes involved in the biosynthetic pathway.

The ubiquitin/26S proteasome system (UPS) pathway is one of the mechanisms involved in post-translational regulation of gene expression [14,15] and is capable of recognizing specific proteins and directing their degradation [16]. The UPS comprises enzymes that act in a concerted catalytic process, including ubiquitin (Ub), ubiquitin-activating enzyme (E1), ubiquitin-conjugating enzyme (E2), ubiquitin ligase (E3), the 26S proteasome (26S), and deubiquitinases (DUB) [17,18]. Ubiquitin is named after a protein that is ubiquitously present in eukaryotes, typically containing 76 conserved amino acids, including two glycines at the C-terminus. Ubiquitin is attached to substrates through a three-step enzymatic cascade, involving E1 activation of Ub, E2 binding of Ub, and E3 recognition of the substrate [19]. The E3 ubiquitin ligase family is the largest among the three enzymes that catalyze the ubiquitination cascade and exhibits the greatest diversity. It is a family of proteins capable of recognizing modified target proteins and is the determinant of ubiquitination specificity [20]. Previous research has proposed classifications of ubiquitin ligases, and four families (HECT, RING, U-box, and cullin) are generally classified based on the functional domains they possess [21,22].

U-box proteins contain a 70-amino-acid U-box domain and are single proteins that are widely distributed in yeast, plants, and animals [23,24,25]. U-box proteins participate in many cellular processes, such as self-incompatibility and pseudo-self-incompatibility, plant hormone responses, and both abiotic and biotic stresses [26,27,28]. For instance, AtPUB19 negatively regulates ABA and drought responses in *A. thaliana* [29]. OsPUB67 participates in abiotic stress responses and the regulation of transcription-related genes in rice in an ABA-dependent manner, mediating a multifaceted and complex drought stress tolerance mechanism [30]. Overexpression of *TaPUB15-D* can enhance salt tolerance in transgenic rice [31]. Although PUBs have been identified in many plants, and there are numerous reports on the effects of biotic and abiotic stresses on protein levels, few studies have reported the impact and mechanisms of PUBs on secondary metabolites in medicinal plants.

Plants possess defensive strategies when subjected to stress, and the rapid accumulation of ABA is one method of responding to stress. ABA is an important plant hormone that guides seed maturation and controls seed dormancy to ensure seeds germinate under favorable growth conditions [32]. During seedling growth and plant maturation, the accumulation of ABA can protect plants from damage due to drought, salinity, and pathogens [33,34]. There are also studies reporting that ABA treatment and environmental factors can affect changes in plant secondary metabolites [35,36,37]. Therefore, studying the expression of PUB, pathway genes, and related transcription factors in *U. rhynchophylla* tissue culture seedlings treated with ABA will help to more comprehensively understand the accumulation patterns and mechanisms of secondary metabolites in *U. rhynchophylla*.

The *UrPUB* genes with high transcript level values in the roots of *U. rhynchophylla* were selected, and their expression was analyzed using qRT-PCR. At the same time, high-performance liquid chromatography (HPLC) was used to measure the content changes of four MIAs, and association analyses were performed between the content of MIAs and pathway genes, pathway genes and transcription factors, and transcription factors and UrPUBs to infer the potential regulatory mechanisms of UrPUB involvement in the biosynthesis of MIAs in *U. rhynchophylla*.

## 2. Results

### 2.1. Identification of UrPUB Family Genes in U. rhynchophylla

To identify the *UrPUB* genes, after initially screening the candidate genes, 73 UrPUBs were ultimately identified by checking the integrity of their domains. They were named *UrPUB1~UrPUB73* based on their positions on the chromosome (Table S1). The molecular weight range of the 73 UrPUB proteins ranged between 23.5 (UrPUB62) and 209 kDa (UrPUB21), and the predicted amino acid content was between 207 (UrPUB62) and 1873 (UrPUB21). The theoretical isoelectric points of the 73 UrPUB proteins ranged from 5.01 (UrPUB2) to 9.38 (UrPUB35), and 41 of them were predicted to be acidic proteins with a theoretical isoelectric point between 5.01 and 6.89. There were 22 UrPUB proteins with a theoretical isoelectric point between 8.08 and 9.38 and therefore designated as alkaline proteins. In addition, 10 UrPUB proteins were predicted as electrically neutral, with a theoretical isoelectric point range between 7.01 and 7.92. The predicted subcellular localization indicated that most UrPUB proteins are located in the nucleus, whereas UrPUB25 is predicted to be located in the cytoplasm, and UrPUB66 and UrPUB73 were predicted to be located in both the cytoplasm and nucleus (Table S2).

### 2.2. Phylogenetic Relationship of PUB Proteins in U. rhynchophylla and A. thaliana

**Figure 2:**
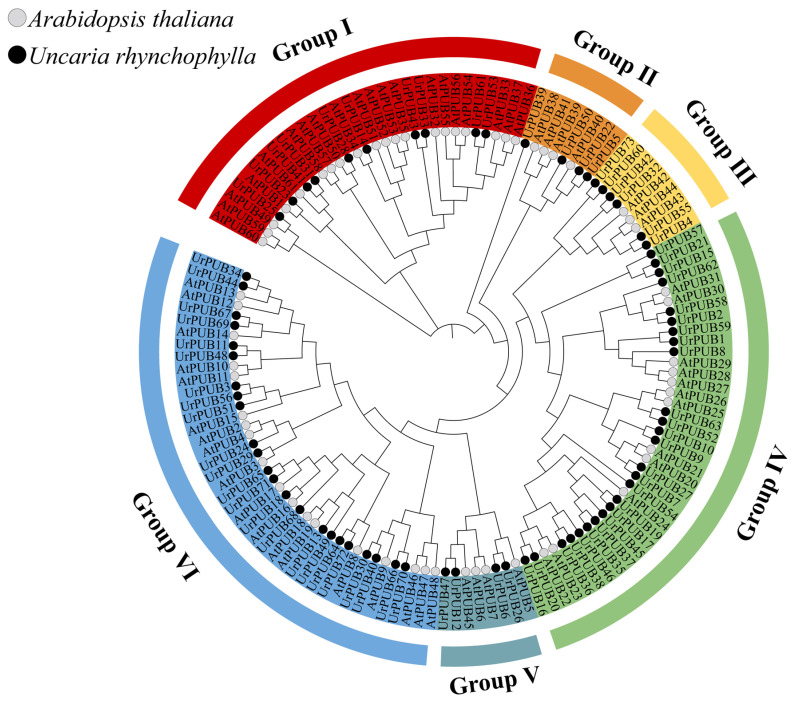
Evolutionary relationship analysis of the PUB protein family in *U. rhynchophylla* and *A. thaliana*. Phylogenetic tree of the *PUB* gene family in *U. rhynchophylla* and *A. thaliana*. Multiple sequence alignments for PUB domain sequences of 73 UrPUBs and 61 AtPUBs were conducted via MEGA 7.0. The phylogenetic tree was established using the neighbor-joining method with MEGA7.0 software, employing a 1000-bootstrap value. The *PUB* genes were categorized into six distinct groups (Group I–Group VI), each identified by a unique color. Black circles represent UrPUBs, gray circles represent AtPUBs.

In order to investigate the evolutionary relationship of PUBs in *U. rhynchophylla*, a neighbor-joining (NJ) phylogenetic tree was constructed using the PUB protein sequences from *A. thaliana* and *U. rhynchophylla* (73 members from *U. rhynchophylla*, 61 members from *A. thaliana*) (Figure 2). They were divided into six subgroups; in group I, there were 20 AtPUBs and 10 UrPUBs. In this group, the protein sequences of UrPUB14, UrPUB 28, UrPUB 33, UrPUB 43, UrPUB 53, and UrPUB 61 all contain protein kinase domains, while UrPUB31 and 41 contain tetratrico peptide repeats (TPRs), and the remaining two UrPUBs contained only a U-box domain. Group II had the same number of AtPUBs and UrPUBs (four). The sequences of UrPUB5, UrPUB 22, and UrPUB 50 contain armadillo (ARM) repetitions. Group III contained three AtPUBs and six UrPUBs, among which UrPUB73 and UrPUB60 contained ARM repetitions. Group IV contained 12 AtPUBs and 26 UrPUBs, among which UrPUB15, UrPUB21, and UrPUB57 contained Trp Asp (WD) repeat profiles and Trp Asp (WD) repeat circular profiles; the remaining UrPUBs in this group had only a U-box domain. Group V had four members each from *A. thaliana* and *U. rhynchophylla*, and these proteins only contained the U-box domain. Group VI included 18 AtPUBs and 23 UrPUBs. In this group, except for UrPUB30, UrPUB40, UrPUB66, and UrPUB70, which only had U-box domains, all other UrPUBs contain different numbers of ARM repetitions.

The number of PUB genes identified in *U. rhynchophylla* is higher than that in *A. thaliana*. Although in group I, UrPUBs had much fewer genes than *A. thaliana*. The number of UrPUBs in groups II and V was the same as the number of AtPUBs, but there was gene expansion of UrPUBs in group III, IV and VI, as *A. thaliana* had fewer members than *U. rhynchophylla*.

### 2.3. Gene Structure and Motif Analysis of UrPUB Genes

**Figure 3:**
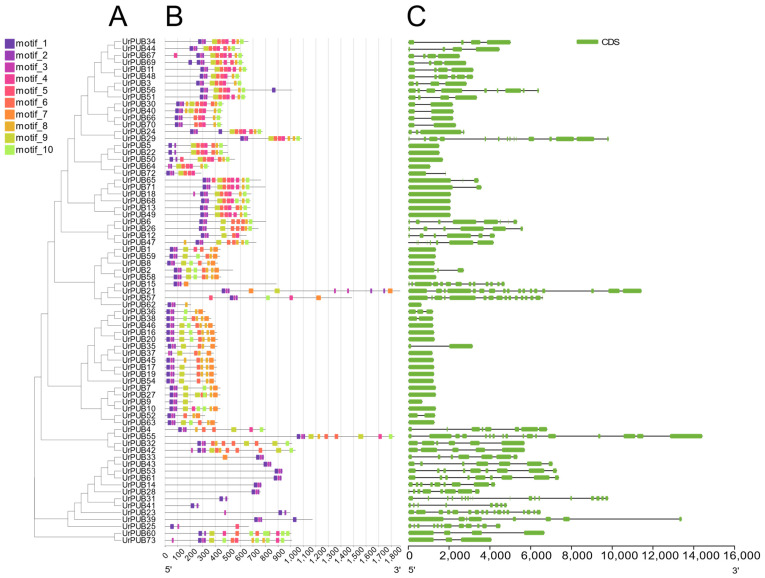
The conserved motifs and gene structure analysis of the *UrPUB* gene family. (**A**) A neighbor-joining (NJ) phylogenetic tree of 73 *UrPUB* genes was constructed by Mega 7.0 with 1000 bootstraps. (**B**) Distribution of 10 motifs in all of the UrPUBs. A total of 10 motifs were predicated and named motif 1–10. (**C**) The gene structure of 73 *UrPUB* genes.

All 73 identified UrPUB protein sequences were uploaded to the MEME website for online analysis to determine conserved motifs [38,39], and ten conserved motifs were identified (Figure 3). The number of motifs contained in these protein sequences was variable, with UrPUB73 containing up to 14 conserved motifs, while UrPUB31 and UrPUB34 had only two motifs. Among these conserved motifs, motif1, motif2, and motif8 have the highest occurrence, appearing 74 times in different sequences; motif7 has the lowest occurrence, appearing only 26 times in all sequences. All UrPUBs contain at least two of the motifs, motif1, motif2, and motif3, with most UrPUBs containing all three motifs, which typically occurred together. There are 54 sequences that start with the order motif1, motif3, motif2, indicating the significant role of these three motifs in UrPUBs. Thirty-four sequences end with motif10, twenty-four sequences end with motif7, and motif8 usually appears adjacent to either motif7 or motif10 at the end of the sequence. Some sequences contain the same motifs; UrPUB34 and UrPUB44 share the same motifs, as do UrPUB3, UrPUB48, and UrPUB56; UrPUB66 and UrPUB70; UrPUB5 and UrPUB22; UrPUB6 and UrPUB26; UrPUB2, UrPUB58, UrPUB16, UrPUB20, and UrPUB46; and UrPUB43, UrPUB53, UrPUB61, UrPUB14, and UrPUB28.

By comparing the coding sequences and genomic DNA sequences of UrPUBs, the structure of *UrPUB* genes can be analyzed. The gene structures of *UrPUB*s are diverse, with intron numbers ranging from 1 to 17. *UrPUB31* had the most introns at 17, while there are 26 *UrPUBs* without any introns. *UrPUB14* and *UrPUB61* not only share the same motifs but also have similar patterns of intron and exon distribution; however, the introns of *UrPUB14* are longer.

### 2.4. Chromosome Localization and Collinearity Analysis of UrPUBs

**Figure 4:**
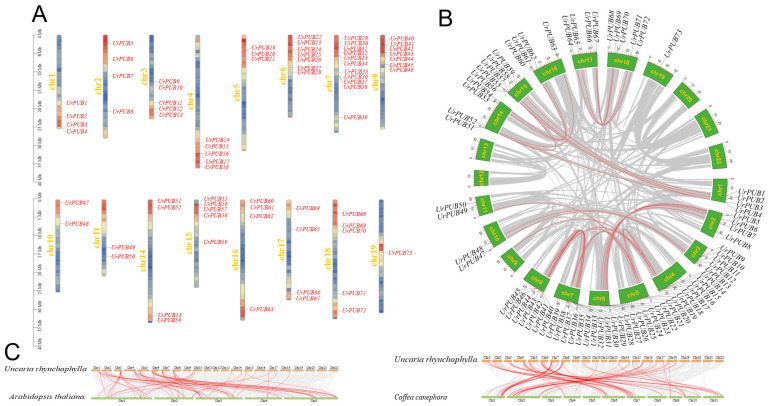
The chromosomal location and collinearity relationship of *UrPUB*s. (**A**) *UrPUB*s are marked on chromosomes. The scale bar on the left indicates the length of *U. rhynchophylla* chromosomes (Mb). (**B**) The chromosomal location and collinearity relationship of *UrPUB* genes in *U. rhynchophylla*. Chromosomes 1–22 are represented by green boxes. Gray lines in the background indicate all *U. rhynchophylla* genome synteny blocks. The red lines represent the gene pairs of segmental duplication. (**C**) Syntenic relationship of *PUB* genes among *U. rhynchophylla*, *A. thaliana*, and *C. canephora*. Identified collinear *UrPUB*s are connected by red lines.

*U. rhynchophylla* has a total of 22 chromosomes, but only 16 of them contain UrPUBs (absent from chr9, chr12, chr13, chr20, chr21, and chr22). On the 16 chromosomes with *UrPUB*s, they are unevenly distributed, with a maximum of 11 UrPUBs on chr 7 but only one on chr19 (*UrPUB73*) (Figure 4A). This uneven distribution of 73 *UrPUB* genes could indicate their functional diversity in performing different biological processes. The intraspecific collinearity analysis showed that there are no tandem duplicated gene pairs and only segmental duplication gene pairs in *UrPUB*s, with a total of 75 collinear gene pairs involving 58 *UrPUB*s. There were 59 collinear gene pairs between *U. rhynchophylla* and *A. thaliana*, involving 37 *UrPUB*s, and 69 gene pairs between *U. rhynchophylla* and *C. canephora*, involving 63 *UrPUB*s. Segmental duplication events are likely the primary cause for the expansion of the *UrPUB*s gene family (Figure 4B,C). Ka/Ks analysis of all segmental duplicate pairs revealed that all analyzable pairs had Ka/Ks < 1, indicating purifying selection. The remaining pairs showed saturated synonymous substitution (Ks not calculable), consistent with their ancient origin. Detailed values are provided in Supplementary Table S3.

### 2.5. Prediction of Cis-Acting Elements in Promoter Regions and Gene Ontology Analysis

**Figure 5:**
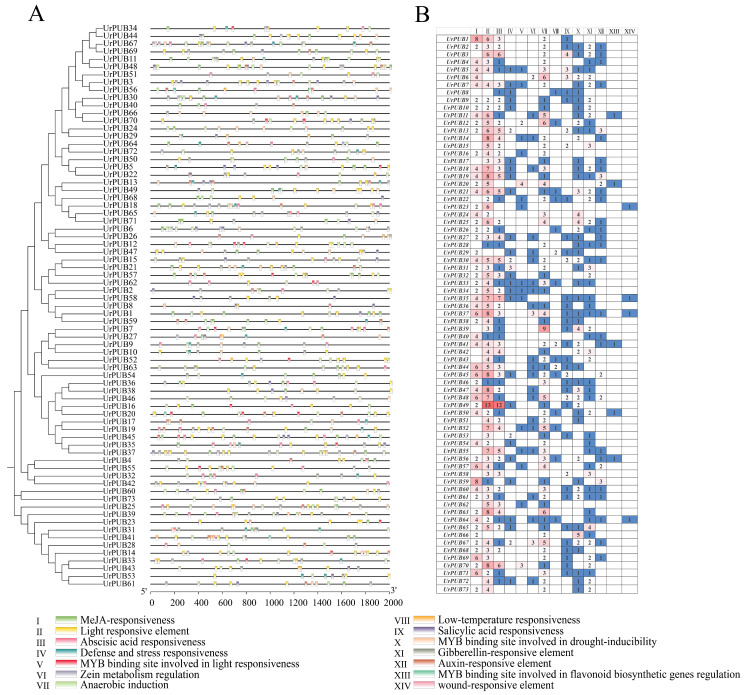
The *cis*-acting elements draft of the putative promoters of 73 *UrPUB* genes. (**A**) The distribution pattern of 14 *cis*-acting elements of the putative promoters of *UrPUB* genes. Different elements are represented by boxes of different colors. (**B**) Statistical chart of *cis*-acting elements in the promoter regions of 73 *UrPUB* genes. The closer to blue, the fewer *cis*-acting elements; the closer to red, the more *cis*-acting elements.

In order to gain further insight into the function of UrPUBs, DNA sequences were extracted from the 2000 bp promoter regions of the *UrPUB* genes and submitted to PlantCARE for the identification of *cis*-acting elements. Fourteen types of *cis*-acting elements related to stress, hormones, plant growth, and development were identified within the promoter regions of the 73 *UrPUB* genes. As shown in Figure 5A,B, *UrPUB* genes are predicted to have a variety of biological functions, and several common hormone-related *cis*-acting elements were found in the promoter regions of the *UrPUB* genes, including ABA, salicylic acid (SA), gibberellin (GA), auxin, and methyl jasmonate (Me-JA). The ABA responsiveness element was found 162 times in total within the promoter regions of 62 *UrPUB* genes, indicating that most *UrPUB* genes are likely to be sensitive to ABA responses. Furthermore, 190 Me-JA-responsiveness elements were identified within the promoter regions of 57 *UrPUB* genes, and 309 light-responsive elements were found in the promoter regions of 69 *UrPUB* genes, indicating that these *UrPUB* genes may be extensively involved in the processes by which *U. rhynchophylla* responds to a variety of abiotic stresses. Flavonoids can help plants cope with abiotic stresses through multiple pathways [40,41], and many studies have shown that R2R3MYB is widely involved in the regulation of flavonoid biosynthesis [42,43]. Therefore, when counting *cis*-acting elements, MYB binding sites were also considered. There were 79 MYB binding sites involved in drought-inducibility in the promoter region of 50 *UrPUB* genes, five MYB binding sites involved in the regulation of flavonoid biosynthetic genes in the promoter region of five *UrPUB* genes, and 22 MYB binding sites involved in light responsiveness in the promoter region of 16 *UrPUB* genes. It should be noted that these in silico predictions do not prove actual responsiveness; they serve as a starting point for future functional studies.

To gain a deeper understanding of the biological function of the *UrPUB* genes, GO annotation was conducted (Figure S1). The results show that 185 GO terms covering biological processes (167), cellular components (four) and molecular functions (14) were notably enriched. Notably, molecular functions were diverse and included key activities such as ubiquitin protein ligase activity, ubiquitin–protein transferase activity, catalytic activity, transferase activity, receptor serine/threonine kinase binding, etc. The biological processes involved mainly include the response to the nitrogen compound, the macromolecule metabolic process, post-translational protein modification, protein modification by small protein conjugation, etc. These UrPUBs are only annotated as potentially involved in the composition of cyclosol, cycloplasm, nucleus, and plasma membrane.

### 2.6. HPLC Quantification of MIAs in the Roots of U. rhynchophylla at Various Time Points Following ABA Treatment

**Figure 6:**
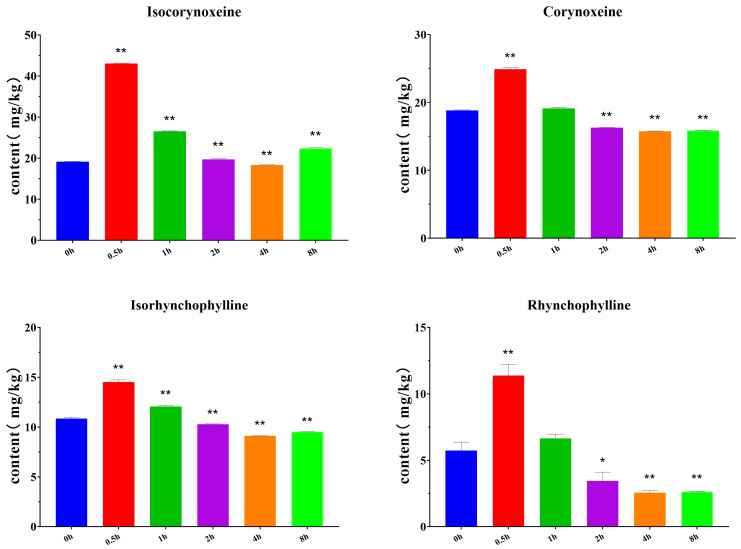
Corynoxeine, Isocorynoxeine, Isorhynchophylline, and Rhynchophylline accumulation in *U. rhynchophylla* roots after ABA treatment for 0 h, 0.5 h, 1 h, 2 h, 4 h, and 8 h. The data are shown as the mean ± SD from three independent biological replicates (*n* = 3). * represents *p* < 0.05, and ** represents *p* < 0.01.

The content of MIAs in the roots of *U. rhynchophylla* was determined at six time points after ABA treatment. As shown in Figure 6, the content variation trends of Isocorynoxeine, Corynoxeine, Isorhynchophylline, and Rhynchophylline were basically consistent, all showing a trend of rising first and then falling. The accumulation of the four MIAs reached a peak at 0.5 h and then gradually decreased and stabilized after ABA treatment.

### 2.7. Expression Analysis of UrPUB Genes Under ABA Stress Treatment

**Figure 7:**
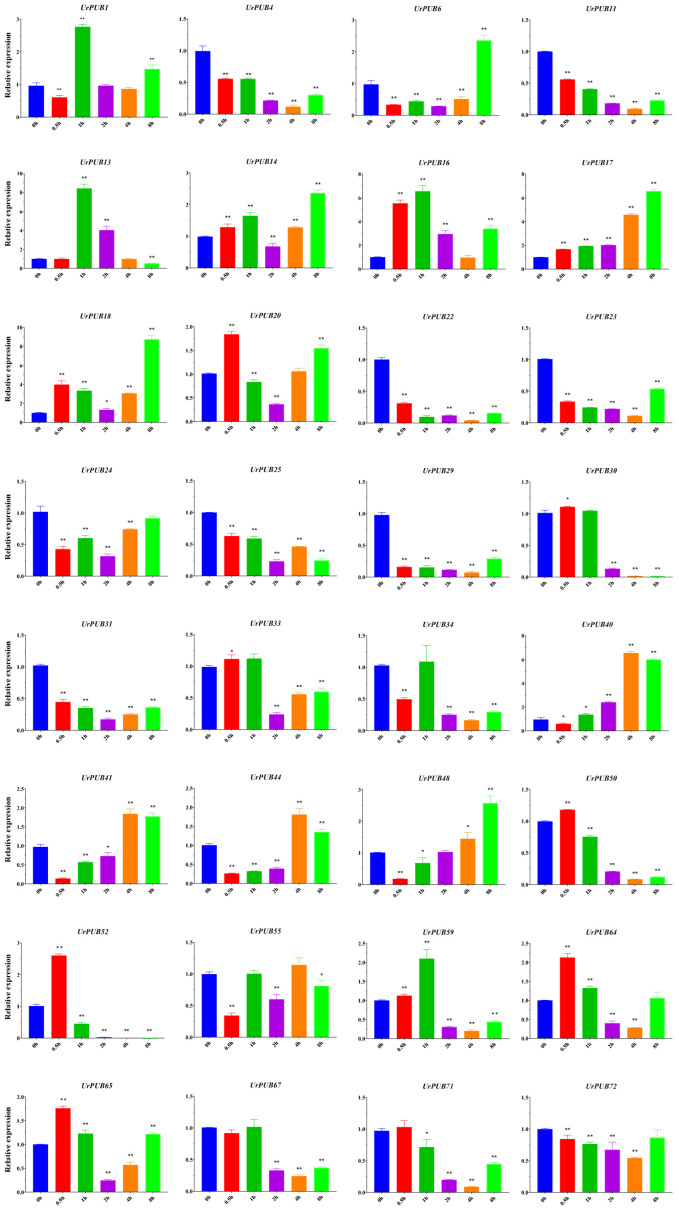
Expression analysis of 32 selected *UrPUB* genes in *U. rhynchophylla* roots after ABA treatment for 0 h, 0.5 h, 1 h, 2 h, 4 h, 8 h. The final results are expressed as mean + standard deviation from three biological replicates (*n* = 3). * *p* < 0.05, ** *p* < 0.01.

In order to investigate the changes in the expression levels of *UrPUB* genes and key enzyme genes under ABA treatment, qRT-PCR was used to measure the relative expression levels of *UrPUB*s in *U. rhynchophylla* at six time points (0 h, 0.5 h, 1 h, 2 h, 4 h, 8 h) after ABA treatment. The results shown in Figure 7 indicate a decreasing trend for *UrPUB4*, *UrPUB11*, *UrPUB22*, *UrPUB25*, *UrPUB29*, *UrPUB30*, *UrPUB31*, and *UrPUB67.* Only *UrPUB17* and *UrPUB40* showed an increasing trend. *UrPUB13*, *UrPUB50*, *UrPUB52*, and *UrPUB59* exhibited an initial increase followed by a decrease. *UrPUB6*, *UrPUB23*, *UrPUB24*, *UrPUB33*, *UrPUB41*, *UrPUB44*, *UrPUB48*, *UrPUB71*, and *UrPUB72* showed an initial decrease followed by an increase. *UrPUB1* and *UrPUB34* demonstrated an initial decrease, then an increase, and finally another decrease. *UrPUB14*, *UrPUB16*, *UrPUB18*, *UrPUB20*, *UrPUB64*, and *UrPUB65* exhibited an initial increase, then a decrease, and finally another increase. *UrPUB55* initially decreased after ABA treatment and then recovered, and under the continuous stimulation of ABA, it was in a process of continuous reduction and recovery. *UrPUB* genes with consistent trends may have similar functions in response to ABA stimulation.

### 2.8. Analysis of the Expression of Transcription Factors That May Be Involved in Regulating Pathway Genes After ABA Treatment

**Figure 8:**
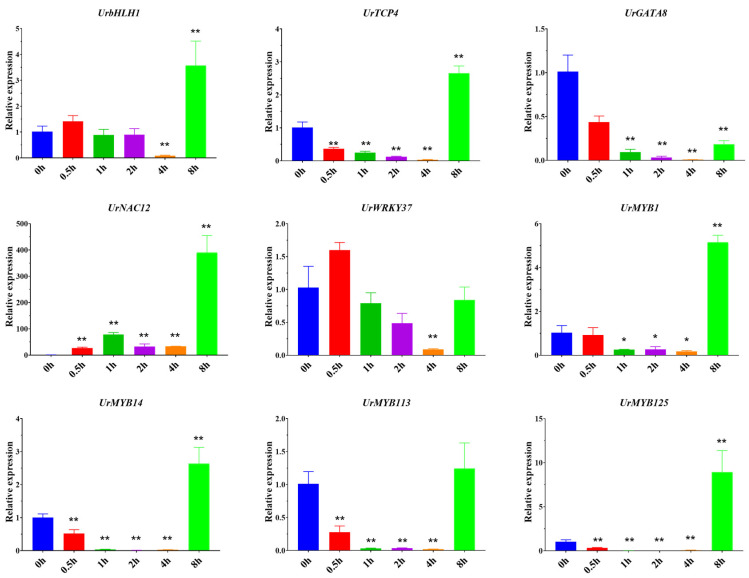
Expression analysis of nine selected transcription factors in *U. rhynchophylla* roots after ABA treatment for 0 h, 0.5 h, 1 h, 2 h, 4 h, 8 h. The final results are expressed as mean + standard deviation from three biological replicates (*n* = 3). * represents *p* < 0.05, and ** represents *p* < 0.01.

As shown in Figure 8, a total of nine transcription factors were selected. UrbHLH1, UrWRKY37, UrTCP4 and UrGATA8 have been reported to possibly participate in the regulation of MIAs biosynthesis [10,11,12,13]. UrNAC12, UrMYB1, UrMYB14, UrMYB113, and UrMYB125 are potential transcription factors that may be involved in regulating the biosynthesis of MIAs in *U. rhynchophylla*, as identified through multi-omics co-expression correlation analysis by our research group (not yet published). The relative expression levels of these nine transcription factors were measured in the roots of *U. rhynchophylla* at six time points after ABA treatment. The expression levels of *UrTCP4*, *UrMYB1*, *UrMYB14*, *UrMYB113*, and *UrMYB125* showed a trend of a slow decrease from 0 h to 4 h followed by a sharp increase at 8 h, while *UrGATA8* initially decreased sharply before slightly rising at 8 h. The expression trends of all *UrMYB* genes were consistent. *UrbHLH1* and *UrWRKY37* exhibited a trend of initial increase followed by a decrease, while the expression level of *UrNAC12* first increased, then decreased, and finally rose sharply.

### 2.9. Co-Expression Correlation Analysis

**Figure 9:**
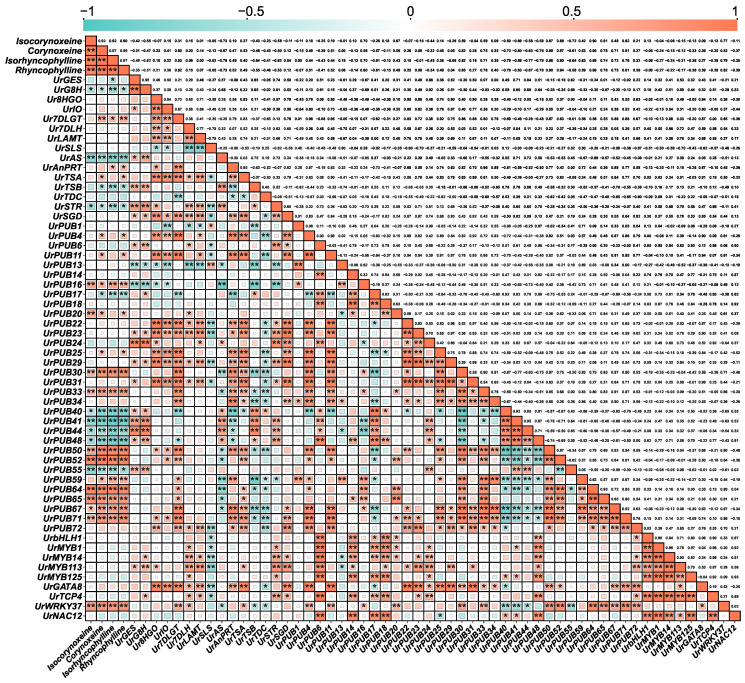
Co-expression correlation analysis was performed to relate the content of MIAs with the relative expression levels of *UrPUB* genes. The heatmap displays Spearman’s rank correlation coefficients of expression levels between genes. In the upper right part of the figure, the numerical values of the correlation coefficients are displayed, whereas in the lower left part, the area size of the rectangles and the intensity of the color represent the absolute value of the correlation coefficient, with red indicating a positive correlation and green indicating a negative correlation. Asterisks denote statistically significant correlations after Benjamini–Hochberg correction for multiple testing: “*” FDR < 0.05, “**” FDR < 0.01. A total of 1770 pairwise comparisons were performed (60 variables: 4 MIA contents, 15 pathway genes, 32 *UrPUB* genes, and nine transcription factors). The expression data used for correlation were derived from three independent biological replicates per time point.

In previous studies, the changes in expression levels of 15 pathway genes in the roots of *U. rhynchophylla* after ABA treatment at different time points were examined, and thus, this data can be directly used for our co-expression correlation analysis [44]. In Figure 9, *UrAS* and *UrSTR* are significantly negatively correlated with changes in four MIA contents, while *Ur7DLGT* is significantly positively correlated with four MIA contents. *UrMYB125*, *UrMYB1*, *UrTCP4,* and *UrMYB14* are all significantly positively correlated with *UrSTR* and *Ur7DLH. UrTCP4* and *UrMYB14* are also significantly positively correlated with *UrLAMT*, *UrSGD*, and *UrG8H. UrWRKY37* is significantly positively correlated with *Ur7DLH* and *Ur7DLGT*, while it shows a significant negative correlation with *UrTSB* and *UrAS*. *UrGATA8* exhibits a significant positive correlation with the expression levels of *UrLAMT*, *Ur7DLH*, *UrSGD*, *UrTSA*, *Ur8HGO*, *UrIO*, *UrAnPRT*, and *Ur7DLGT*, but displays a significant negative correlation with *UrSLS* and *UrTDC*. The expression trend of *UrSLS* is solely negatively correlated with transcription factors, showing a significant negative correlation with *UrMYB1*, *UrbHLH1*, *UrTCP4*, *UrMYB14*, *UrMYB113*, and *UrGATA8*. In the correlation analysis between transcription factors and *UrPUB* genes, *UrWRKY37* is significantly positively correlated with *UrPUB64*, *UrPUB65*, *UrPUB20*, *UrPUB72*, *UrPUB71*, *UrPUB33*, *UrPUB50*, *UrPUB30*, *UrPUB67*, *UrPUB52*, *UrPUB11*, and *UrPUB4* and negatively correlated with *UrPUB40*, *UrPUB41*, *UrPUB44*, and *UrPUB55. UrGATA8* is negatively correlated with *UrPUB17* and *UrPUB40*, *UrNAC12* is negatively correlated with *UrPUB25*, and *UrPUB13* is negatively correlated with *UrMYB14* and *UrMYB113*. *UrPUB6*, *18*, and *UrPUB14* are significantly positively correlated with *UrTCP4*, *UrMYB14*, *UrbHLH1*, *UrMYB125*, and *UrMYB1*.

## 3. Discussion

U-box E3 ubiquitin ligases play a significant role in plant response to abiotic stress, and *U. rhynchophylla* produces important pharmacological medicinal products that are known to be responsive to abiotic stress. To understand the potential function of PUBs in *U. rhynchophylla* and its potential role in MIA biosynthesis, it is important to first identify and bioinformatically characterize all the PUBs in its genome. The Pfam database was utilized for HMMER analysis of the *U. rhynchophylla* genome, and 73 *UrPUB* genes were identified. The number of PUB genes in *U. rhynchophylla* is higher than that found in potato (66) [45] and *Sorghum bicolor* L. (68) [46] but lower than that in banana (91) [47] and maize (85) [48].

Phylogenetic analysis divided the UrPUBs into six groups, with the phylogenetic tree showing some resemblance to the groupings found in banana [47]. All of these UrPUBs possessed at least one U-box domain, and additionally, there were ARM domains, protein kinase domains, Trp-Asp (WD40) repeats and TPRs. Twenty-one UrPUB proteins contained varying numbers of ARM repeats. Six UrPUB proteins contained a protein kinase domain, all of which were classified into the first group on the phylogenetic tree. Two UrPUB proteins contained TPRs, structurally similar to AtCHIP [49], suggesting that these proteins may be involved in signal transduction through phosphorylation and affect protein degradation in chloroplasts [50,51]. Three UrPUB proteins contained Trp-Asp (WD40) repeats, potentially involved in transcriptional regulation and signal transduction [52,53]. These different structures might lead to distinct biological functions of UrPUB proteins. The prevalence of ARM domains is noteworthy, aligning with findings from other studies, and this domain likely plays a crucial role in facilitating interactions with substrates, leading to their ubiquitination [54,55]. Analysis of the gene structures revealed that some *UrPUB* genes lack introns, while the majority contain several introns, which can protect the coding sequences from mutations. Additionally, the presence of genes without introns also reflects the integrity of the *UrPUB* genes structure. Among the motifs identified from all *UrPUB* genes, motifs 1, 2, and 3 exhibited high conservation. Analyzing the *UrPUB* gene structure and motifs aids in understanding their evolutionary history and functions.

Previous research has shown that PUB genes are capable of responding to ABA signaling [56]. The *cis*-acting elements in the promoter regions of *UrPUB* genes were analyzed to predict their potential biological functions. There were a total of 162 ABREs (ABA-responsive elements), with *UrPUB49* containing the highest number, which suggests a potential role in ABA signaling transduction. The promoter regions of the *UrPUB* genes contain numerous *cis*-acting elements, including not only ABREs but also a variety of other hormone response elements such as salicylic-acid-responsive elements, MeJA-responsive elements, gibberellin-responsive elements, and auxin-responsive elements. Notably, there are 190 MeJA-responsive elements, raising the possibility that *UrPUB* genes could respond to hormones other than ABA, such as salicylic acid, MeJA, gibberellin, and auxin. Previous studies have shown that MYB transcription factors are extensively involved in the gene regulation of plant responses to abiotic stresses. For instance, overexpression of *CgMYB1* from *Chenopodium glaucum* in *A. thaliana* enhances tolerance to salt and cold stress [57]. The overexpression of the *Betula platyphylla* MYB transcription factor gene *BplMYB46* can influence abiotic stress tolerance [58], and the overexpression of the *Zea mays* MYB transcription factor ZmMYB3R improves drought and salt resistance in transgenic plants [59]. Therefore, MYB binding sites were analyzed and found to be distributed across many *UrPUB* genes. These UrPUBs may act in concert with MYB transcription factors to respond to biotic and abiotic stresses. Hence, it is likely that *UrPUB* genes play a crucial role in various biological processes during the growth and development of *U. rhynchophylla*.

ABA can induce changes in the content of secondary metabolites within plants. In *Salvia miltiorrhiza,* SmbZIP1 expression in hairy roots responds to ABA stimulation and regulates the expression of biosynthetic genes such as *SmC4H1*, thereby controlling the biosynthesis of tanshinones and salvianolic acids [60]. Out of the 73 *UrPUB* genes, 62 (85%) contain 162 ABREs, and these ABREs are commonly distributed in other species; for example, in *S. miltiorrhiza*, 53 out of 60 *SmPUB* genes have ABREs [61]. In the plant hormone response cis-acting elements of *ZmPUB* genes, 73% have ABREs [48]. Therefore, we investigated how *UrPUB* genes respond to ABA. Initially, HPLC was used to measure the content changes of four major MIAs in *U. rhynchophylla* after ABA treatment. It was found that ABA can significantly affect the accumulation of MIAs in *U. rhynchophylla* (Figure 7), with four MIAs exhibiting highly similar patterns of content change, peaking at 0.5 h after ABA treatment and then gradually decreasing. The content of MIAs in *U. rhynchophylla* is also regulated by transcription factors. UrGATA8, UrbHLH1, UrWRKY37, and UrTCP4 may all be involved in regulating the biosynthesis of MIAs in *U. rhynchophylla*. Existing studies suggest that E3 ubiquitin ligase might regulate the content of secondary metabolites in plants by degrading specific transcription factors. The RING3-type E3 ubiquitin ligase mediates the degradation of AsWRKY44, promoting the biosynthesis of sesquiterpenes induced by injury in *Aquilaria sinensis* [62,63].

To understand whether a regulatory mechanism exists in *U. rhynchophylla* that involves ubiquitin ligase-mediated degradation of transcription factor proteins, qRT-PCR was employed to analyze the expression of *UrPUB* genes with transcripts per million (TPM) values greater than 10 in roots (the complete TPM values for all 73 *UrPUB* genes in roots, with the 32 genes having TPM > 10 clearly indicated, have been provided as Supplementary Table S4), as well as the expression of the known *U. rhynchophylla* transcription factors after ABA treatment. By integrating the changes in expression levels of pathway genes in *U. rhynchophylla* following ABA treatment, a correlation analysis was performed between the metabolite content and pathway genes, pathway genes and transcription factors, and transcription factors with *UrPUB* genes. Initially, a co-expression correlation analysis was performed between the MIA content and pathway genes. Since pathway genes are involved in the biosynthesis of MIAs, the focus was placed on those that showed a positive correlation with alkaloid content. It was observed that *Ur7DLGT* had a strong positive correlation with alkaloid content, leading to a focus on these two pathway genes in subsequent investigations. Considering that transcription factors regulate pathway genes, potential transcription factors that might respond to ABA signals and regulate the biosynthesis of MIAs could also be deduced by analyzing the correlation between them. In the correlation analysis, *UrWRKY37* and *UrGATA8* displayed significant positive correlations with *Ur7DLGT*. Therefore, it is hypothesized that UrWRKY37 and UrGATA8 could potentially respond to ABA stimuli and affect the biosynthesis of MIAs. Previous studies have indicated that PUB proteins can regulate the biosynthesis of plant hormones such as ABA and ethylene, and ubiquitinate transcription factors to indirectly control fruit ripening and coloration. *AtPUB18*, *AtPUB19*, and *AtPUB44* have been identified as capable of directly interrupting ABA biosynthesis in *A. thaliana* [64]. In apple, the ubiquitin E3 ligase MdPUB29 ubiquitinates MdbHLH3 to regulate ethylene biosynthesis [65]. An attempt was made to understand whether UrPUB proteins might possess similar indirect regulatory mechanisms. Consequently, a correlation analysis was conducted between *UrPUB* genes and the mentioned transcription factors. Since PUB proteins do not regulate transcription but rather ubiquitinate proteins to facilitate their degradation, focus was placed on *UrPUB* genes that were negatively correlated with *UrGATA8* and *UrWRKY37*. The correlation analysis revealed that *UrPUB17* and *UrPUB40* were significantly negatively correlated with *UrGATA8*, while *UrPUB40*, *UrPUB44*, *UrPUB44*, and *UrPUB55* showed significant negative correlations with *UrWRKY37*. It is tempting to speculate that these negative correlations reflect ubiquitination and degradation of the TFs by UrPUBs, but we emphasize that such gene–expression-level correlations are inherently indirect and cannot establish causality. Alternative explanations are equally plausible: for example, these TFs might repress UrPUB transcription, or UrPUBs and TFs might be antagonistically regulated by an unknown common upstream signal; moreover, a negative correlation does not distinguish whether a TF is a substrate of the PUB or instead acts as a regulator of PUB expression. Therefore, the proposed ubiquitination model remains a hypothesis without direct supporting evidence. The gene expression correlations reported here should be viewed as hypothesis-generating observations only, not as evidence for physical interaction, ubiquitination, or degradation. Future studies measuring TF protein stability (e.g., cycloheximide chase assays) and direct ubiquitination status (e.g., ubiquitination assays followed by immunoblotting), as well as protein–protein interaction assays (e.g., Co-IP or pull-down) and proteasome inhibitor (MG132) experiments, are required to test whether these TFs are genuine substrates of UrPUBs. Additionally, a limitation of our experimental design is the absence of a solvent-treated mock time-course. Thus, we cannot rule out non-ABA factors (e.g., culture duration, handling, circadian rhythms) contributing to the observed changes. Our results should be interpreted as changes detected after ABA treatment, not as strictly ABA-specific, and future mock time-course studies are needed.

## 4. Materials and Methods

### 4.1. Plant Material and ABA Treatment

The *U. rhynchophylla* plant materials used in this study were identified by Professor Wei Shugen and collected from the College of Horticulture, Hunan Agricultural University, Changsha City, Hunan Province. The method for obtaining *U. rhynchophylla* tissue culture seedlings is consistent with that mentioned in previous studies [12]. ABA treatment was administered to *U. rhynchophylla* tissue culture seedlings with uniform growth, with sampling conducted at 0 h, 0.5 h, 1 h, 2 h, 4 h, and 8 h post-treatment. ABA was added to 1/2 MS liquid medium to a final concentration of 100 μM. The root system of each *U. rhynchophylla* tissue culture seedling was evenly divided into two parts. One part was dried to a constant weight and then ground into powder for the detection of MIA content, while the other part was immediately frozen using liquid nitrogen and stored in an −80 °C freezer for RNA extraction and cDNA synthesis.

### 4.2. UrPUB Genes Search and Identification

In order to identify PUB genes in the *U. rhynchophylla* genome, a search was conducted in the *U. rhynchophylla* genome database [66]. A search was conducted in the Pfam database based on the U-box domain (PF04564). The HMMER program can be used to identify potential members of the PUB gene family in *U. rhynchophylla*. All sequences obtained were submitted to the SMART (http://smart.embl.de/, accessed on 26 February 2025) website to confirm the U-box domain [67,68]. The integrity of the domains was confirmed using the NCBI CDD (https://www.ncbi.nlm.nih.gov/Structure/bwrpsb/bwrpsb.cgi, accessed on 25 March 2025) and Expasy (https://prosite.expasy.org/ accessed on 25 March 2025) websites [69]. The physicochemical properties of UrPUB proteins, including the number of amino acids, relative molecular weight, theoretical isoelectric point, and instability index, were predicted using Expasy (https://web.expasy.org/protparam/, accessed on 26 March 2025). Subcellular localization was predicted using Ploc2 (http://www.csbio.sjtu.edu.cn/bioinf/Cell-PLoc-2/, accessed on 3 April 2025) [70].

### 4.3. Multiple Sequence Alignment and Phylogenetic Tree Construction

The PUB gene sequences of *A. thaliana* were obtained from the TAIR database (https://www.arabidopsis.org/, accessed on 11 April 2025). The ClustalW software (version 2.1) was used to compare 73 UrPUB protein sequences with 61 AtPUB protein sequences. In MEGA software (version 11.0.13), the neighbor-joining method was employed to construct the phylogenetic tree, with bootstrapping repeated 1000 times.

### 4.4. Analysis of Conserved Motifs and Gene Structure

Conserved motif analysis of UrPUB amino acid sequences was conducted using the MEME online tool (https://meme-suite.org/meme/doc/meme.html, accessed on 13 April 2025), with the number of conserved motifs set to 10. The TBtools software (version 2.210) was used to extract exon and intron information corresponding to *UrPUB* genes from the *U. rhynchophylla* genome for gene structure analysis [71]. The conservation of motifs and gene structures were ultimately visualized using TBtools software.

### 4.5. Chromosomal Location, Gene Duplication, and Synteny Analysis

The chromosomal location information of *UrPUB*s was extracted from the genome using TBtools software, and the intraspecific synteny within *U. rhynchophylla,* the synteny between *U. rhynchophylla* and *A. thaliana*, and the synteny between *U. rhynchophylla* and *C. canephora* were analyzed. The visualization of these results was completed in TBtools software. Based on the results of the synteny analysis, segmental duplication and tandem duplication events involving *UrPUB* genes could be identified.

### 4.6. Promoter cis-Element Analysis and Gene Ontology Analysis

The method for obtaining the 2000 bp sequence upstream of the promoter of *UrPUB* genes was consistent with previous studies. The promoter sequences were uploaded to the PlantCare website (http://bioinformatics.psb.ugent.be/webtools/plantcare/html/, accessed on 16 April 2025) to predict *cis*-acting elements [72,73]. The raw data was filtered and processed to select candidate *cis*-acting elements for statistical analysis. Based on the GO annotation results in the genome database, the GO annotation results and *cis*-acting element diagrams of *UrPUB* genes were visualized using TBtools. Quantitative statistics and visualization were completed in GraphPad Prism 9.0 software.

### 4.7. Determination of Four MIAs in the Roots of U. rhynchophylla by HPLC

Dried root powder of *U. rhynchophylla* tissue-cultured seedlings was sieved through a 60-mesh screen. A 0.1 g aliquot was extracted with 1 mL of 80% (*v*/*v*) HPLC-grade methanol in an ultrasonic bath at 50 °C for 60 min. The extract was centrifuged at 12,000 rpm for 5 min, and the supernatant was filtered through a 0.22 μm membrane.

Chromatographic separation used a reversed-phase column (GL-Sciences GL-C18, 250 mm × 4.6 mm, 5 μm, Tokyo, Japan). Mobile phase A: 0.2% ammonia water; mobile phase B: acetonitrile. Gradient elution: 0–40 min, 28–55% B; 40–60 min, 55–63% B; 60–60 min, 63–28% B; 60–70 min, 28–28% B. Detection wavelength: 254 nm; column temperature: 30 °C; flow rate: 1.0 mL/min; injection volume: 20 μL.

Standard solutions and calibration curves: stock solutions (0.5 mg/mL in 80% methanol) of rhynchophylline, isorhynchophylline, corynoxeine, and isocorynoxeine were prepared. A seven-point calibration curve was generated by serial two-fold dilution of the mixed stock solution. Figure S2 shows the calibration curves for the four alkaloids, with the regression equations, concentration ranges, and correlation coefficients (all R^2^ > 0.99). The MIA content in each sample was calculated using the respective standard curve and expressed as mg per kilogram dry weight. The method of liquid chromatography was consistent with that of previous studies [10].

### 4.8. RNA Extraction, cDNA Synthesis, and qRT-PCR

Total RNA was extracted using the SteadyPure Plant RNA extraction Kit (Accurate Biology, Hunan, China). The integrity of the RNA was assessed by electrophoresis on a 1% agarose gel. The concentration of the extracted RNA was measured using Micro Drop (BIO-DL, Shanghai, China). For reverse transcription, 1 µg of the extracted RNA was used to synthesize cDNA according to the EvoM-MLV RT Mix Kit (Accurate Biology, Hunan, China). qRT-PCR primers for *UrPUB* genes were designed using Beacon Designer 7.0 software, and the primers used in this study are shown in Table S5. Reactions were performed in a 96-well plate in an ABI7300 (Applied Biosystems, Foster City, CA, USA). *UrSAM* was used as the reference gene in this study [74,75]. The reaction protocol and system were consistent with those of previous studies. For each of the six time points (0, 0.5, 1, 2, 4, 8 h), three independent biological replicates were analyzed. Technical triplicates were averaged per biological replicate. Correlation analyses were performed using these 18 independent data points (6 time points × 3 replicates). The obtained Ct values were used to calculate the relative expression levels using the 2^−ΔΔCT^ method. Statistical tests were performed using GraphPad Prism 9.0 software. For each time point, unpaired two-tailed Student’s *t*-tests were used to compare relative expression levels with the 0 h control. Differences with *p* < 0.05 were considered statistically significant. Correlation analysis between MIA content and gene expression levels was conducted on the chiplot website (https://www.chiplot.online/, accessed on 31 October 2025).

## 5. Conclusions

This study identified 73 *UrPUB* genes in the genome of *U. rhynchophylla*. Following ABA treatment, the contents of four MIAs showed consistent temporal changes. Based on co-expression analysis, UrPUB17, UrPUB40, UrPUB41, UrPUB44, and UrPUB55 emerged as candidate regulators that may be involved in MIA biosynthesis under ABA stimulation, possibly through ubiquitination of UrGATA8 and UrWRKY37. However, this proposed model remains a hypothesis that requires experimental validation (e.g., protein–protein interaction and ubiquitination assays). These results provide new insights into the potential biological functions of PUB genes under ABA stimulation and offer testable hypotheses for future research.

## Data Availability

The 73 *UrPUB* sequences and the relevant transcription factor sequences used in this study are provided in the Supplementary Materials. The genome of *U. rhynchophylla* discussed in this study is currently under investigation and cannot be disclosed at this time. For any requests, please contact the corresponding authors.

## Supplementary Materials

The following supporting information can be downloaded at: https://www.mdpi.com/article/10.3390/ijms27125198/s1.
