# Supplementary material for: Genome-Wide Identification and Expression Analysis of the U-Box E3 Ubiquitin Ligase Gene Family Related to Monoterpene Indole Alkaloids Biosynthesis in Uncaria rhynchophylla
Source: Int J Mol Sci. 2026 Jun 9;27(12):5198. doi: 10.3390/ijms27125198 (PMC13299250; doi:10.3390/ijms27125198)
Supplement: Supplementary file 1 [file ijms-27-05198-s001.zip › ijms-4329165-supplementary.pdf]

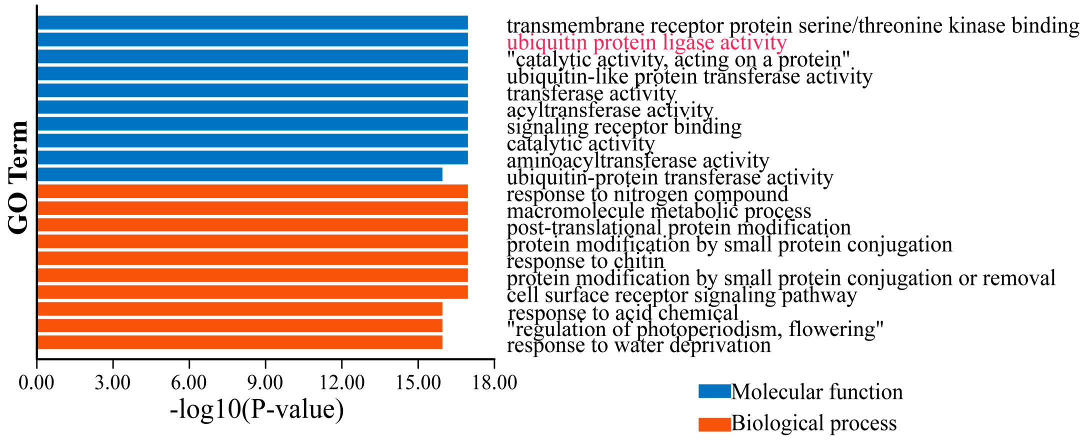

Figure S1. Gene Ontology Analysis (Partial Results)

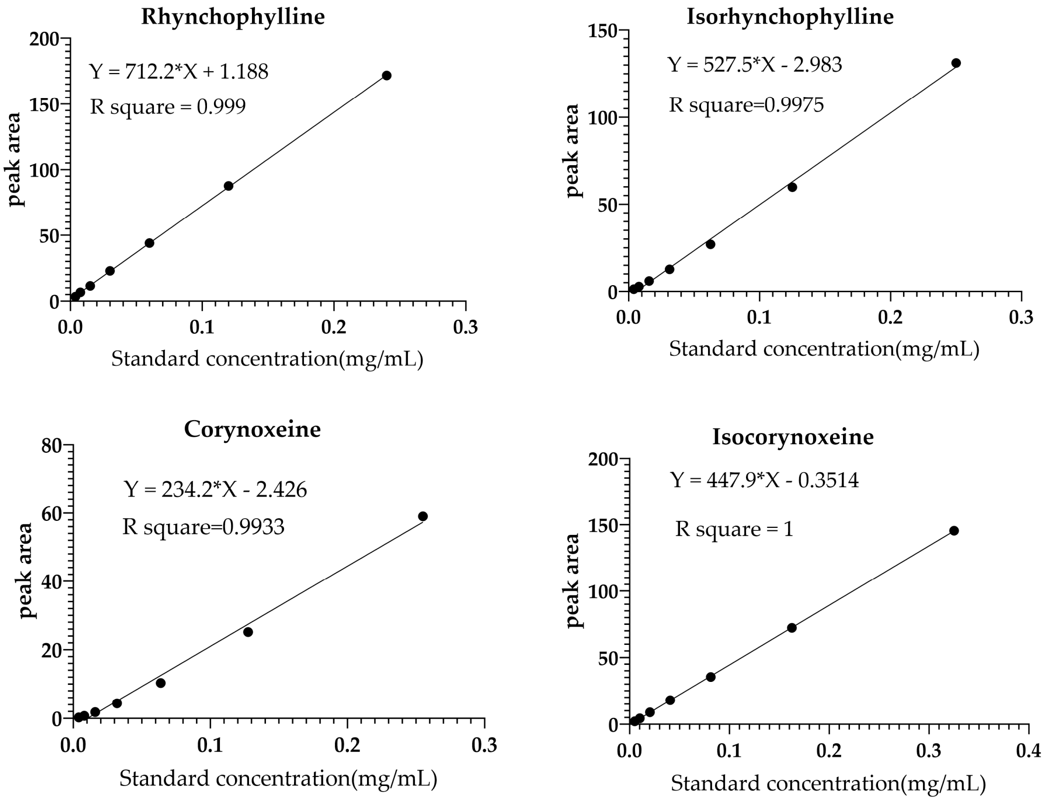

Figure S2. Standard curves of rhynchophylline, isorhynchophylline, corynoxine, and isocorynoxine

Table S1: The chromosomal location of UrPUB genes

Gene Name	Gene number	Chromosome number	Start position	Termination position
UrPUB1	g32668.t1	chr1	18406199	18407530
UrPUB2	g33088.t1	chr1	22024899	22027597
UrPUB3	g26983.t1	chr1	25261955	25264791
UrPUB4	g26998.t2	chr1	25385336	25392121
UrPUB5	g19785.t1	chr2	2251893	2253386
UrPUB6	g6719.t2	chr2	6423698	6429008
UrPUB7	g13892.t1	chr2	11127566	11128894
UrPUB8	g28521.t1	chr2	20815186	20816454
UrPUB9	g18982.t1	chr3	12584561	12585226
UrPUB10	g18971.t1	chr3	12991666	12992994
UrPUB11	g20994.t1	chr3	18406736	18409905
UrPUB12	g2143.t1	chr3	19975216	19979430
UrPUB13	g2306.t1	chr3	21100585	21102630
UrPUB14	g24781.t1	chr4	29255450	29259684
UrPUB15	g24653.t1	chr4	30245067	30249765
UrPUB16	g24389.t1	chr4	32206874	32208118
UrPUB17	g24388.t1	chr4	32216774	32218018
UrPUB18	g17992.t1	chr4	35907412	35909478
UrPUB19	g42859.t1	chr5	4209093	4210334
UrPUB20	g42858.t1	chr5	4220922	4222190
UrPUB21	g42535.t1	chr5	6494732	6506147
UrPUB22	g34086.t1	chr6	1300540	1302045
UrPUB23	g33971.t1	chr6	2007847	2014310
UrPUB24	g33680.t1	chr6	3932993	3935716
UrPUB25	g33637.t1	chr6	4202346	4206841
UrPUB26	g33403.t1	chr6	5813821	5819414
UrPUB27	g18579.t1	chr6	8876532	8877860
UrPUB28	g18475.t1	chr6	10134894	10138358
UrPUB29	g34959.t1	chr7	2432967	2442774
UrPUB30	g34792.t1	chr7	3485606	3487764
UrPUB31	g34791.t1	chr7	3491724	3501502
UrPUB32	g34616.t1	chr7	4659181	4664858
UrPUB33	g34475.t1	chr7	5553324	5558651
UrPUB34	g34470.t1	chr7	5597426	5602423
UrPUB35	g20873.t1	chr7	11369957	11373092
UrPUB36	g20874.t1	chr7	11387316	11388505
UrPUB37	g37126.t1	chr7	11431132	11432298
UrPUB38	g37124.t1	chr7	11464956	11466150
UrPUB39	g29856.t1	chr7	22337933	22351321
UrPUB40	g9133.t1	chr8	2932221	2934414
UrPUB41	g9132.t1	chr8	2938501	2943312
UrPUB42	g8972.t1	chr8	3905786	3911477
UrPUB43	g8820.t1	chr8	4793983	4801034
UrPUB44	g8814.t1	chr8	4851769	4856220

UrPUB45	g8445.t1	chr8	7966630	7967853
UrPUB46	g8444.t1	chr8	7981001	7982200
UrPUB47	g15704.t1	chr10	839471	843632
UrPUB48	g15056.t1	chr10	6495239	6498389
UrPUB49	g16953.t1	chr11	12994055	12996103
UrPUB50	g41406.t1	chr11	15397467	15399140
UrPUB51	g1919.t1	chr14	267092	270433
UrPUB52	g1617.t1	chr14	2065682	2066975
UrPUB53	g16343.t1	chr14	31597410	31604668
UrPUB54	g43898.t1	chr14	32676913	32678130
UrPUB55	g22907.t1	chr15	1177198	1191595
UrPUB56	g22897.t2	chr15	1286842	1293225
UrPUB57	g22807.t1	chr15	1804275	1810855
UrPUB58	g22437.t1	chr15	4278779	4280125
UrPUB59	g36222.t1	chr15	11514565	11515884
UrPUB60	g4104.t1	chr16	342382	349037
UrPUB61	g4254.t1	chr16	1401422	1408784
UrPUB62	g4595.t1	chr16	4430608	4431231
UrPUB63	g25942.t1	chr16	29774032	29775291
UrPUB64	g14525.t1	chr17	2332326	2333381
UrPUB65	g35551.t1	chr17	7991611	7995031
UrPUB66	g10727.t1	chr17	25707081	25709260
UrPUB67	g10596.t1	chr17	26795278	26797780
UrPUB68	g40817.t1	chr18	3844636	3846684
UrPUB69	g40374.t1	chr18	7017270	7020081
UrPUB70	g11203.t1	chr18	8440774	8443087
UrPUB71	g9701.t1	chr18	25432392	25435959
UrPUB72	g10383.t1	chr18	30058172	30059991
UrPUB73	g38661.t1	chr19	14397518	14401578

Table S2: Physicochemical properties of UrPUB proteins

Gene Name	Gene ID	Protein length (aa)	Molecular weight (Da)	Theoretical isoelectric point(PI)	Instability index	Aliphatic index	GRAVY	Subcellular localization
UrPUB1	g32668.t1	443	48885.42	8.16	46.25	97.88	-0.023	Nucleus.
UrPUB2	g33088.t1	542	59366.65	5.01	47.57	105.18	0.111	Nucleus.
UrPUB3	g26983.t1	611	67652.45	7.2	32.78	106.82	-0.106	Nucleus.
UrPUB4	g26998.t2	800	88058.61	5.05	47.35	105.32	-0.039	Nucleus.
UrPUB5	g19785.t1	497	54034.67	6.87	54.17	95.67	-0.182	Nucleus.
UrPUB6	g6719.t2	805	90288.55	5.47	47.11	98.78	-0.07	Nucleus.
UrPUB7	g13892.t1	442	49839.69	6.39	39.87	96.76	-0.172	Nucleus.
UrPUB8	g28521.t1	422	47236.16	8.69	46.49	100.83	-0.022	Nucleus.
UrPUB9	g18982.t1	221	25096.98	7.59	46.19	89.64	-0.267	Nucleus.
UrPUB10	g18971.t1	442	49431.66	8.13	43.39	102.17	-0.093	Nucleus.
UrPUB11	g20994.t1	655	71581.6	6.81	42.46	98.9	-0.212	Nucleus.
UrPUB12	g2143.t1	650	72268.55	5.51	49.82	88.52	-0.306	Nucleus.
UrPUB13	g2306.t1	681	75974.35	6.33	34.03	110.1	0.113	Nucleus.
UrPUB14	g24781.t1	772	86334.81	6.58	57.3	89.27	-0.33	Nucleus.
UrPUB15	g24653.t1	889	99623.55	5.38	47.43	99.46	-0.083	Nucleus.
UrPUB16	g24389.t1	414	46163.94	8.61	54.01	102.44	-0.019	Nucleus.
UrPUB17	g24388.t1	414	46321.65	8.42	41.01	96.88	-0.07	Nucleus.
UrPUB18	g17992.t1	688	75431.41	8.89	53.68	105.48	0.006	Nucleus.
UrPUB19	g42859.t1	413	46324.26	8.97	41.35	106	0.04	Nucleus.
UrPUB20	g42858.t1	422	46973.62	8.49	49.28	101.66	-0.069	Nucleus.
UrPUB21	g42535.t1	1873	209134.46	5.4	48.86	94.7	-0.14	Nucleus.
UrPUB22	g34086.t1	501	54981.7	8.22	52.55	89.68	-0.276	Nucleus.
UrPUB23	g33971.t1	996	112961.35	5.14	47.85	95.08	-0.178	Nucleus.
UrPUB24	g33680.t1	777	83990.16	5.44	44.22	97.95	-0.161	Nucleus.
UrPUB25	g33637.t1	668	73515.23	8.08	32.85	71.83	-0.475	Cytoplasm.
UrPUB26	g33403.t1	745	82523.45	6.15	48.59	100.27	-0.088	Nucleus.
UrPUB27	g18579.t1	442	49876.68	6.64	40.6	98.55	-0.192	Nucleus.

UrPUB28	g18475.t1	762	86781.69	6.88	43.98	82.22	-0.55	Nucleus.
UrPUB29	g34959.t1	1090	120404.29	5.83	45.13	86.86	-0.408	Nucleus.
UrPUB30	g34792.t1	467	51831.88	7.92	37.82	100.69	-0.188	Nucleus.
UrPUB31	g34791.t1	505	57114.11	9.01	52.76	91.82	-0.322	Nucleus.
UrPUB32	g34616.t1	1012	111246.32	5.84	46.75	100.05	-0.107	Nucleus.
UrPUB33	g34475.t1	789	87937.51	6.42	45.29	88.42	-0.278	Nucleus.
UrPUB34	g34470.t1	665	72501.97	5.35	43.97	98.87	-0.223	Nucleus.
UrPUB35	g20873.t1	417	47540.02	9.38	45.39	105.52	-0.046	Nucleus.
UrPUB36	g20874.t1	322	36346.26	8.2	48.81	95.4	-0.149	Nucleus.
UrPUB37	g37126.t1	388	44238.22	9.26	48.72	105.34	-0.012	Nucleus.
UrPUB38	g37124.t1	369	41200.53	9.2	48.84	106.5	-0.034	Nucleus.
UrPUB39	g29856.t1	1175	131669.61	6	49.24	90.38	-0.38	Nucleus.
UrPUB40	g9133.t1	460	51305.31	6.44	33.12	102.65	-0.149	Nucleus.
UrPUB41	g9132.t1	276	31765.11	5.49	42.58	81.7	-0.54	Nucleus.
UrPUB42	g8972.t1	1040	114618.92	6.2	44.36	101.6	-0.115	Nucleus.
UrPUB43	g8820.t1	848	95066.77	7.3	50.02	87.52	-0.267	Nucleus.
UrPUB44	g8814.t1	597	64361.4	5.58	42.8	96.1	-0.2	Nucleus.
UrPUB45	g8445.t1	407	46006.01	8.79	42.21	107.15	0.047	Nucleus.
UrPUB46	g8444.t1	399	44627.15	9.02	40.8	108.52	-0.099	Nucleus.
UrPUB47	g15704.t1	727	80782.27	5.64	50.01	90.17	-0.274	Nucleus.
UrPUB48	g15056.t1	598	65099.23	7.13	43.07	95.62	-0.208	Nucleus.
UrPUB49	g16953.t1	682	76053.42	8.25	41.45	102.83	0.017	Nucleus.
UrPUB50	g41406.t1	557	59123.54	7.59	53.9	97.52	-0.012	Nucleus.
UrPUB51	g1919.t1	645	72075.68	7.85	38.61	105.57	-0.162	Nucleus.
UrPUB52	g1617.t1	320	35234.78	9.09	32.18	101	0.104	Nucleus.
UrPUB53	g16343.t1	937	105139.26	5.83	54.1	81.3	-0.456	Nucleus.
UrPUB54	g43898.t1	405	45579.03	6.68	50.85	106.1	-0.013	Nucleus.
UrPUB55	g22907.t1	1827	199508.1	5.42	50.73	79.04	-0.375	Nucleus.
UrPUB56	g22897.t2	1013	112546.83	7.3	43.65	93.12	-0.341	Nucleus.
UrPUB57	g22807.t1	1488	165825.86	5.93	45.59	90.01	-0.26	Nucleus.
UrPUB58	g22437.t1	448	48583.84	6.2	47.13	112.05	0.204	Nucleus.

UrPUB59	g36222.t1	439	48394.98	8.73	53.21	98.38	-0.034	Nucleus.
UrPUB60	g4104.t1	1005	112677.64	6.03	46.91	108.42	-0.12	Nucleus.
UrPUB61	g4254.t1	930	104885.92	5.87	58.27	80.43	-0.463	Nucleus.
UrPUB62	g4595.t1	207	23503.43	6.32	47.18	90.39	-0.227	Nucleus.
UrPUB63	g25942.t1	419	45228.22	7.01	41.35	106.63	0.184	Nucleus.
UrPUB64	g14525.t1	351	38090.05	8.94	41.56	110	0.048	Nucleus.
UrPUB65	g35551.t1	764	83820.44	6.72	37.95	103.98	0.12	Nucleus.
UrPUB66	g10727.t1	451	49819.57	5.63	44.21	104.55	-0.164	Cytoplasm. Nucleus.
UrPUB67	g10596.t1	623	68305.65	5.96	40.95	105.28	-0.161	Nucleus.
UrPUB68	g40817.t1	682	74815.43	8.11	50.67	108.71	-0.021	Nucleus.
UrPUB69	g40374.t1	625	68736.96	5.8	46.96	103.98	-0.19	Nucleus.
UrPUB70	g11203.t1	457	50565.85	6.18	42.18	111.07	-0.052	Nucleus.
UrPUB71	g9701.t1	800	88234.06	6.89	46.75	96.51	0.041	Nucleus.
UrPUB72	g10383.t1	290	31086.82	7.57	39.1	107.59	0.131	Nucleus.
UrPUB73	g38661.t1	1012	112336.5	5.52	45.36	108.02	-0.086	Cytoplasm. Nucleus.

Table S3: Ka/Ks analysis of segmentally duplicated UrPUB gene pairs in Uncaria rhynchophylla

Seq_1	Seq_2	Ka	Ks	Ka_Ks	Note
g32668.t1	g36222.t1	0.1201159198543232	0.4743879964532326	0.25320185323484423	
g33088.t1	g22437.t1	0.05664692313373796	0.4418021540362565	0.1282178518511461	
g26983.t1	g1919.t1	0.2788849114536911	2.404089603402067	0.1160043748199054	
g26983.t1	g22897.t2	0.09148509562734734	0.4206541455886671	0.21748292887812218	
g19785.t1	g34086.t1	0.0950090131615777	0.47596254450962505	0.19961447441093003	
g6719.t2	g33403.t1	0.165556649168559	0.4332907660091131	0.38209133947958857	
g13892.t1	g18579.t1	0.08729826957356465	0.499556101101729	0.17475168330651084	
Seq_1	Seq_2	Ka	Ks	Ka_Ks	Note
g24653.t1	g42535.t1	0.10821193710034893	0.371343668945608	0.2914064413905468	
g24389.t1	g42859.t1	0.46284781280355547	NaN	NaN	High Sequence Divergence Value (pS>=0.75)
g24389.t1	g20873.t1	0.5605955267485421	NaN	NaN	High Sequence Divergence Value (pS>=0.75)
g24389.t1	g8445.t1	0.46450785589522436	NaN	NaN	High Sequence Divergence Value (pS>=0.75)
g42859.t1	g20873.t1	0.39877416754242145	2.1098199045310224	0.18900862897634965	
g42859.t1	g8445.t1	0.351830776570574	1.9252227447626562	0.1827480885147906	
Seq_1	Seq_2	Ka	Ks	Ka_Ks	Note
g34792.t1	g9133.t1	0.07267330777443551	0.3661619737483971	0.19847311568287515	
g34791.t1	g9132.t1	0.06939948945908782	0.32685357616311367	0.21232592977491108	
g34616.t1	g8972.t1	0.10760800287126066	0.44549576983523476	0.24154663221843642	
g34475.t1	g8820.t1	0.10627263150452909	0.39705955951083366	0.26764909434608253	
g34470.t1	g8814.t1	0.07715120286246697	0.40241483209156226	0.19172057466538064	
g20873.t1	g8445.t1	0.13153711125931478	0.6024408987083771	0.21834027460839406	
g15704.t1	g2143.t1	0.07314692656028118	0.43363660702085327	0.16868254519103273	
Seq_1	Seq_2	Ka	Ks	Ka_Ks	Note
g15056.t1	g20994.t1	0.10194085776011969	0.4394220613524372	0.23198848379703518	
g1919.t1	g22897.t2	0.2678265748293591	1.9540959197838386	0.13705907274960485	
g1617.t1	g25942.t1	0.0623343224500411	0.6511677023547997	0.09572698741756266	
g16343.t1	g4254.t1	0.10724959912060254	0.36466892761564507	0.29410128200898383	
g14525.t1	g10383.t1	0.060878311925796944	0.4517189796377681	0.13477032108461562	
g35551.t1	g9701.t1	0.10839154650280508	0.4339749621867244	0.24976451626757204	

g10727.t1	g11203.t1	0.10272291110111298	0.39138178492661957	0.2624621662461184
g10596.t1	g40374.t1	0.06014515155134791	0.29341619988269013	0.20498238193867405

Table S4: TPM values of 73 UrPUB genes in different tissues

Gene_id	R1_fpkm	R2_fpkm	R3_fpkm	L1_fpkm	L2_fpkm	L3_fpkm	S1_fpkm	S2_fpkm	S3_fpkm
UrPUB1	42.55297537	44.03979316	42.53672878	13.01988958	13.42694779	13.10305826	11.22064931	10.06454451	10.23499938
UrPUB2	8.625267987	7.961366161	8.955291637	16.18206828	15.78453769	14.96054611	16.60062041	15.09979561	15.91881083
UrPUB3	0	0	0	0.034348372	0.03279838	0.05796427	0.02928227	0.032597002	0.031597412
UrPUB4	16.91319378	16.14031446	17.91869789	17.05690708	16.41172359	17.09888437	19.18809754	21.13741652	21.01706139
UrPUB5	8.244084649	5.827366547	7.606805188	5.360829013	3.990337898	4.273991969	6.837233655	6.088962813	6.290549732
UrPUB6	21.40196755	19.29920458	19.35811639	32.7347232	33.04159958	33.64571819	26.92476209	28.24810654	28.39427521
UrPUB7	1.304664473	0.632496296	0.698897516	1.043942388	0.996833847	0.800770504	2.589002151	2.161556479	2.226226655
UrPUB8	9.894263956	8.37464827	6.243037401	3.876250297	4.55548569	3.270664776	3.601096188	2.735373925	3.702947715
UrPUB9	0	0	0	0	0	0	0	0	0
UrPUB10	0	0	0	0.189807707	0.045310629	0	0	0	0
UrPUB11	20.90206354	21.81402866	19.71164237	19.3228441	18.54268437	19.84604716	22.073118	22.10851884	20.22195803
UrPUB12	1.836854796	1.567916262	2.433921493	3.358226064	2.466680039	2.779079571	1.596625895	1.348344179	1.633746346
UrPUB13	14.64056766	13.99805454	14.60734388	8.507114626	9.065049144	8.296369895	21.1265138	23.19625579	20.72697534
UrPUB14	21.88934474	22.29242604	21.4167068	32.17087164	32.82248066	31.80281295	35.84149853	34.22103234	34.14727765
UrPUB15	2.978284622	3.395632	2.885342733	5.881213123	4.691126556	5.918998653	5.255410739	5.626169232	5.388459246
UrPUB16	24.34806359	22.90757884	21.63550937	134.6370096	138.0415074	133.8614285	78.41947148	79.31675243	80.09966659
UrPUB17	11.57373437	13.64809434	11.80517651	11.59965207	13.30112633	11.2833388	15.24343251	15.67106745	13.55962942
UrPUB18	16.7769626	14.05917435	14.3003394	30.90635585	30.64787297	33.59487954	14.90361296	13.84005294	13.44371261
UrPUB19	0.481397936	0.531772679	0.527897016	0.456982685	1.01817581	0.471274718	0.865736679	0.48186872	0.840765906
UrPUB20	45.08957431	47.69291218	45.72486703	83.58785896	90.68263701	83.0245674	69.31051016	68.8088027	74.42467752
UrPUB21	0.159524076	0.277675421	0.213806742	1.155380984	0.985421564	1.088453931	0.688523985	0.681301686	0.402437043
UrPUB22	16.11858379	19.01727729	16.97893469	14.57246779	15.99411061	16.39442019	11.5306813	11.56427334	9.784371363
UrPUB23	36.7413334	36.39454739	36.64410106	36.81346175	30.90416704	34.7980967	32.60605734	33.39567723	32.66253268
UrPUB24	28.92137321	30.43258366	28.77004808	33.42317323	27.1934829	30.77768638	32.52454751	33.12927967	29.97579011
UrPUB25	11.17145434	9.363794991	12.05996119	15.89944542	15.69203948	16.25234958	13.95621878	12.19624856	14.19248039
UrPUB26	3.87376918	4.453515186	3.954981796	18.85145464	20.58384148	17.95105273	5.885500764	5.8297072	5.702782152
UrPUB27	5.038704172	5.782823276	4.193385097	2.182788629	2.763948393	2.882773816	2.872174261	5.223761491	2.750044691
UrPUB28	8.306291092	7.764273945	7.78146492	6.832579916	6.10333584	5.625648931	5.096726855	4.706272216	4.714019065
UrPUB29	12.95167832	11.42870369	11.75211981	12.2158048	13.79876867	13.78646429	11.87598691	13.22033996	14.53423006

UrPUB30	29.55413019	27.6261485	29.57576869	53.00217985	55.4141252	53.62809688	56.59587054	58.35615532	56.31874016
UrPUB31	15.28219629	16.92882943	15.83691047	16.36828892	18.84286401	17.10610383	16.43325624	16.28278211	16.58601833
UrPUB32	6.374411995	4.860258167	6.130744758	6.723464901	5.607648861	5.410418165	7.430123091	7.109299869	7.788496805
UrPUB33	11.27677711	10.28567078	10.48944734	19.13195613	20.40291757	19.30870549	16.99068506	16.94433407	17.01220634
UrPUB34	9.059928159	10.04650926	8.818563941	13.13565828	12.12678835	11.87140296	12.44545618	13.73611263	15.46245689
UrPUB35	0	0	0	0	0.240102976	0.084866348	0.042872606	0.047725754	0
UrPUB36	0	0.247851269	0.056385285	0	0	0.439308153	0	0	0
UrPUB37	0.102467221	0	0	0.054039084	0.361203758	0.091193145	0	0.051283715	0
UrPUB38	0.161593577	0.054091865	0.246114149	0.113628127	0.434002353	0.383504144	0	0.161751608	0.104527653
UrPUB39	1.694717223	1.514664204	1.192481651	0.715006923	0.819290156	1.101025397	0.716220421	0.67854983	0.707072688
UrPUB40	27.14959048	25.96174833	24.57297626	17.78366459	17.19887307	20.62270659	30.32144127	29.98895867	32.46706001
UrPUB41	29.06739825	26.29997233	32.41421079	18.51687243	22.39146616	18.56949218	36.42376114	34.7133355	38.0469337
UrPUB42	9.859640142	8.44009758	9.359903136	27.68498563	19.06994248	26.95488902	9.726439322	10.0417553	8.953631927
UrPUB43	5.023549059	5.398355353	5.362911377	3.788273428	4.09017824	4.429043739	1.98415834	2.138271161	1.731046883
UrPUB44	32.52768821	29.55242995	30.88197541	23.09520187	23.29496743	27.88100781	32.93462114	32.09245675	30.07354972
UrPUB45	0	0.098107794	0.133915052	0.66979325	0.836358701	0.521678432	0.08784681	0.097791005	0.189584469
UrPUB46	1.444915904	0.950664522	1.32040241	6.096149024	6.523597866	4.922036001	2.55370677	2.144556738	2.46554602
UrPUB47	7.665336361	7.450262736	7.004787322	16.57441048	14.64087264	16.83559488	6.227952698	5.89163939	5.737534371
UrPUB48	23.48996899	25.12607751	28.30682503	17.65219595	16.78860938	17.58901102	13.4330825	13.75473747	15.0439549
UrPUB49	4.14354639	3.457761072	3.359836496	11.78787841	11.9906653	12.02380947	16.05783097	15.45126514	16.64790354
UrPUB50	9.143454986	11.33408749	11.16246754	19.36361759	17.69842928	18.49995126	13.74566431	11.01147746	13.65415886
UrPUB51	6.509602984	6.970816927	7.781169322	7.321626617	7.830181769	6.78181961	10.90225149	10.9011236	11.7941496
UrPUB52	16.76344587	15.89896397	17.75855428	86.31135905	107.6166971	94.26615496	68.50080794	70.78606461	65.90452253
UrPUB53	7.351531546	6.102346611	7.10634927	10.9364044	9.308725839	10.58929354	6.457583424	6.720681601	6.411512289
UrPUB54	2.994390017	3.401392378	3.095218831	3.313687256	4.054073703	3.320239081	1.633171731	1.818045579	1.571776657
UrPUB55	18.82931832	19.39026253	21.08023531	22.56130264	17.96547875	21.22823343	23.54486631	22.70889991	23.54558255
UrPUB56	2.656448485	1.922284561	2.677426059	3.832025995	3.954192459	3.528870828	4.426032814	4.692434761	3.752546165
UrPUB57	1.003855333	1.465093958	1.088588171	0.847059914	0.633588055	0.929141169	0.385133631	0.602902233	0.584414179
UrPUB58	9.321322168	8.068000396	6.611645584	10.86173546	10.46100326	10.86345954	12.29307522	12.35172265	12.6620469
UrPUB59	37.59499061	35.11545506	33.27910777	44.62228204	44.88965245	42.97207516	25.41488078	27.61173475	24.69940933
UrPUB60	4.497098927	4.61555235	4.815617209	8.734456339	7.043370689	9.097739963	4.239700124	4.917934912	4.671014562

UrPUB61	1.94803285	2.493687253	2.484404698	7.225333115	5.131343608	6.477553887	2.964334465	2.935620844	2.284788123
UrPUB62	0	0.288663316	0.087559842	0.303190436	0	0	0	0	0
UrPUB63	6.121318609	6.528372907	4.900015512	21.47165789	21.64974237	22.88926223	10.92312336	11.5896311	11.32631785
UrPUB64	20.66592104	24.39205023	23.23121795	37.86205409	38.77663067	37.59048789	36.04514341	34.2879711	30.65451602
UrPUB65	23.88979732	20.48490734	21.569251	29.48464234	27.94422012	31.02247739	40.29240358	39.87265241	40.04023986
UrPUB66	0.3086485	0.35422991	1.00732561	0.697606312	0.754943252	0.863308555	1.665202365	0.794443739	1.36903475
UrPUB67	23.09182579	24.7288241	21.86077377	27.25345137	31.84596592	30.3576718	35.64046448	32.32180772	34.3676538
UrPUB68	3.384868882	4.33685287	3.25317502	4.401218309	4.40833283	4.882237971	5.090227463	4.819392717	4.275226929
UrPUB69	9.710242387	7.321411653	7.244248103	10.77924335	10.16456389	9.548548671	10.10547043	10.26149446	11.39869049
UrPUB70	4.395016001	3.845482782	3.022152783	3.396438999	3.681439172	4.337448618	2.543337778	1.829417752	2.997752674
UrPUB71	21.77108642	21.83798652	21.30469772	24.7478089	25.13461504	25.57592011	27.183159	23.93425689	27.13543097
UrPUB72	13.21809548	13.89287274	14.89540342	16.47022132	17.2445093	18.83420482	24.386999	26.94192593	21.33118449
UrPUB73	2.34121922	2.094257584	2.517021308	2.344912141	2.12020646	2.766492136	1.892912311	2.048108549	2.233465996

Table S5: Primers used in this study

Gene	Primer Sequence (5′ → 3′)	
UrPUB1	F:GCGAAGGCATTAGAAGTC	R:TCCAGAGCACCATAATCC
UrPUB4	F:AGCACAGAACTGAATCCA	R:GAATCATCCTCAACCACAAT
UrPUB6	F:CTCTTATGGTCAATCTGTTCAA	R:AGGCTCATACATTACTCTCAA
UrPUB11	F:GGCTGCTGTAGAAGAGAT	R:TGGAGAGGTTAAGAATAGAAGT
UrPUB13	F:ACTTCTGAGTGGTCTAATGG	R:CTGTTGCTATTGTTACTGGAT
UrPUB14	F:GCTGCTCTCAACCTTCTA	R:GTGCTATGTAATTCTTGTCGTA
UrPUB16	F:GAATTGACACCTAACATTACTCT	R:ATGGATCTCAGTCTTCTCAG
UrPUB17	F:TTCCTCAATACTTCGTCTGT	R:GCCAATGTTCAATACTCTCC
UrPUB18	F:CGTTGTTGAGGATTCTTAGG	R:AGCACTATACTGGTTGAACT
UrPUB20	F:CGGATTGGCGATTGTATC	R:GTGGTTGCTGAGAACTTG
UrPUB22	F:GCTAATGCGGAAGAAGTATT	R:TGTCGGAACTCACATCAT
UrPUB23	F:ATCCTGTCATCTTACCTTCTT	R:CACGCTGCTTCAATTCAT
UrPUB24	F:ATCCATCAAGAGCATCCAT	R:TATCAGCATCCAGCACAT
UrPUB25	F:GCAAGGTCACGCATCAAGGA	R:GAACAGAGGCAGAAGCAGCAT
UrPUB29	F:TCTACTTACTTCCTCCAACAA	R:ACTCTTGCCTTCCTTCTG
UrPUB30	F:TGAAGGAGTTGAAGATTAAGAAG	R:TGGCGACAATAACAGGAT
UrPUB31	F:TTGTTCTTCTTCTTCTTCTCAG	R:CATCATCTTCATAGACCTTCAA
UrPUB33	F:GCTTATATGCTTGGCTTGAA	R:ATGGTTGATGGCTTCTACA
UrPUB34	F:GAGGAGAGTATAGAGAAGATGT	R:CAGCAACTTGAAGGATTAGAA
UrPUB40	F:AAGAGATGGTGCTGTGAA	R:ATGGCAAGTATGGCTAATAAC
UrPUB41	F:GAGAGTGGAGGAGGATTG	R:GAGAGCAAGACCTAGCATA
UrPUB44	F:CAACAGCACTCTTCAACTT	R:CATCCACCATACCACCTT
UrPUB48	F:GCTACGGACAGATATGGT	R:CTTGGATGGAATGGATTAACA
UrPUB50	F:ATTACTCGTCCTCCTCAAG	R:ACCAGCAGAAGATGATGT
UrPUB52	F:TTGACAGGTTGGCAGATT	R:CCGAGCACAGAGAAAGTA
UrPUB55	F:TCATCTGGATTGGCTGTT	R:GTCTCTGTCTGTGTCTACTT
UrPUB59	F:AGAGTTGCGATTAGTGAAGA	R:TCCACAGCACCATTATCC
UrPUB64	F:GTGGTGGAAGTCAACAAG	R:TGAGCAGAGGGTAAATAGAG
UrPUB65	F:TCAAGATGGATGGAGGAAG	R:CTCTAATATGTTCACAAGAAGGAT
UrPUB67	F:ATGGTAATCCTGAAGAACAAC	R:AATCTGGTGAGCATAGTAGT
UrPUB71	F:GCTTCTGTCTTGAATGGATT	R:AATGTCTCTGCTATCTCTTGA
UrPUB72	F:GTGGTGGAAGTCAACAAG	R:CCGATTATCAGCGAAGGA
UrNAC12	F:CATTGCCCTGGGGATCA	R:TTCGCATCCGTTGGTGCT
UrMYB1	F:CCCTCAAACCCACCGTCC	R:TCAGCCGTGTTGGTGGTG
UrMYB14	F:GCGAGGAAACTTCGCTCA	R:TCCAGGCAATCTTCCGGC
UrMYB113	F:GAAGGCTGCTGGCGATCA	R:TGCCACGCTTGAGGTCAG
UrMYB125	F:GGAGCATGGACAGCTGAGG	R:CAGCTCTTGCCGCACCTA
UrbHLH	F:ACAGGCGGTCGTGTTAGG	R:TCGTTGGCTGAGCTGCTC
UrWRKY37	F:CCTGTTGGGGTCCACGTG	R:GGCTTTGTCCCTGCACCA
UrTCP4	F:TAGCAGCCAGATGATTAGG	R:TCCGTTACCAGACAATGAA
UrGATA8	F:TTCCAGCACTATTACTTCCA	R:CCACCTGAACAAGAACCT
